# BASE - 2nd generation software for microarray data management and analysis

**DOI:** 10.1186/1471-2105-10-330

**Published:** 2009-10-12

**Authors:** Johan Vallon-Christersson, Nicklas Nordborg, Martin Svensson, Jari Häkkinen

**Affiliations:** 1 Department of Oncology, Clinical Sciences, Lund University, SE-221 84 Lund, Sweden; 2 CREATE Health Strategic Centre for Translational Cancer Research, Lund University, SE-221 84 Lund, Sweden; 3 Department of Theoretical Physics, Lund University, Sölvegatan 14a, SE-223 62 Lund, Sweden

## Abstract

**Background:**

Microarray experiments are increasing in size and samples are collected asynchronously over long time. Available data are re-analysed as more samples are hybridized. Systematic use of collected data requires tracking of biomaterials, array information, raw data, and assembly of annotations. To meet the information tracking and data analysis challenges in microarray experiments we reimplemented and improved BASE version 1.2.

**Results:**

The new BASE presented in this report is a comprehensive annotable local microarray data repository and analysis application providing researchers with an efficient information management and analysis tool. The information management system tracks all material from biosource, via sample and through extraction and labelling to raw data and analysis. All items in BASE can be annotated and the annotations can be used as experimental factors in downstream analysis. BASE stores all microarray experiment related data regardless if analysis tools for specific techniques or data formats are readily available. The BASE team is committed to continue improving and extending BASE to make it usable for even more experimental setups and techniques, and we encourage other groups to target their specific needs leveraging on the infrastructure provided by BASE.

**Conclusion:**

BASE is a comprehensive management application for information, data, and analysis of microarray experiments, available as free open source software at http://base.thep.lu.se under the terms of the GPLv3 license.

## Background

Microarray techniques produce large amounts of data in many different formats and experiment sizes are growing with more samples analysed in each experiment. Samples are collected over long time and microarray analysis is performed asynchronously and re-analysed as more samples are hybridised. Systematic use of collected data requires tracking of biomaterials, array information, raw data, and assembly of annotations. Particularly for microarray service facilities, where researchers deposit samples for experimentation, information tracking becomes vital for a subsequent data delivery back to the researchers. To meet the information tracking and data analysis challenges involved in microarray experiments we reimplemented the obsolete BASE version 1.2 (BASE1) [1].

BASE (BioArray Software Environment) is a MIAME (Minimum Information About a Microarray Experiment guidelines) [2] compliant application designed for microarray laboratories looking for a single point of storage for all information related to their microarray experimentation. BASE is a multi-user local data repository that features a web browser user interface, laboratory information management system (LIMS) for biomaterials and array production, annotations, hierarchical overview of analysis, and integrates tools like MultiExperiment Viewer (MEV) [3] and GenePattern [4].

The utility of BASE as a local repository managing all data and annotations in experimentation in itself merits the applications existence but on top of the repository capabilities there are analysis tools available for different experimental techniques. The current list of plug-ins is biased towards preprocessing of expression and comparative genomic hybridisation (aCGH) data but there are ongoing efforts to provide SNP and methylation array analysis plug-ins, and more techniques and experimental setups will be supported as needs emerge.

## Implementation

The current BASE is a complete rewrite of BASE1 and introduces major enhancements and extensions to support storage of all data produced in microarray experimentation. Importantly, BASE1 was inherently targeted for 2-channel data. Thus, 1-channel data had to be tweaked into the BASE1 database model because of requirements to store raw data in database tables expecting 2-channel data. BASE now supports any microarray data and the requirement of storing raw data in database tables is removed - raw data can be stored in files or in database tables depending on data type, user needs, and expected utility. BASE accommodates common array platforms such as Illumina, Affymetrix, and Agilent, and has the potential to be extended to support future technologies like ultra high-throughput sequencing gene expression data. BASE supports the inevitable trend in the microarray field to use vendor produced stock or customised arrays but retains the BASE1 array production LIMS feature useful for groups that fabricate in-house spotted arrays.

BASE is written in Java and uses Hibernate [5] for object/relational persistence in a MySQL [6] or PostgreSQL [7] database. BASE is a web application residing in a Tomcat container [8] and features an integrated ftp server to simplify batch import/export of data.

To avoid user interface lock-up during potentially time consuming operations a job handling system was implemented where job servers checks for tasks at regular intervals. Many of the tasks requested by the user in BASE, such as import and normalization of data, are from user perspective seamlessly added to the job queue. Jobs are executed in batch mode on the server side under control of the job queue system. The BASE job handling allows the user to log off or continue to work with other tasks in BASE while the requested tasks are executed. Information about job execution is reported by the job handling system, e.g., current job status and start/stop time, is shown in appropriate views. The operation performed by a job is normally supplied as a plug-in, and the job queue is configurable for tuned execution of jobs.

The BASE programming interface aims at facilitating developers to write extensions and plug-ins. Extensions are additions to the user interface that perform direct actions like generating quality control plots, while more complex and time consuming work is performed by plug-ins that work through a job queue. Some extensions like MEV are Java web start enabled applications where the user can spawn an MEV process for further analysis and visualization. Data are automatically transferred to the MEV process running locally on the user computer and analysis can be stored in BASE or on the local file system. 

BASE development is transparent and can be followed through the project web site at http://base.thep.lu.se. The BASE team uses Trac [9] for project management where Trac provides an enhanced wiki and issue tracking system for software development projects. The BASE development is divided into milestones where short and long term milestones are announcement through the project web site. User and developer talk-back is also provided through the wiki or mailing lists set up for BASE users and developers. Sample code and instructions for extension development are available through the BASE co-project site http://baseplugins.thep.lu.se along with many extensions and plug-ins readily available for download and use.

## Results and Discussion

**Figure 1:**
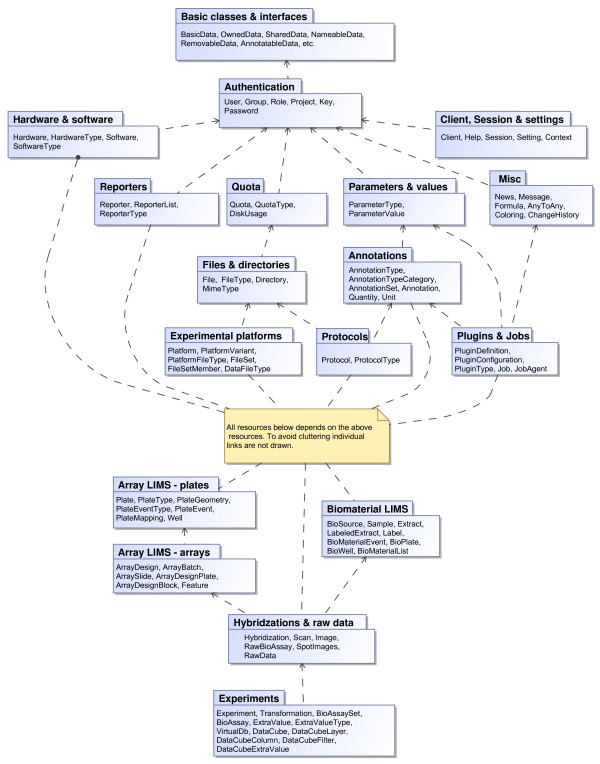
**BASE data layer overview**. The main theme of a microarray experiment is captured in the lower part of the figure where the *Experiments *module groups *BioAssays *into a logical unit and keeps track of analysis made within the experiment. The *Experiments *are connected to *RawData *and physical experimental steps like *Hybridizations*. The *Hybridization *objects connect the array information (*ArrayDesign*, *ArraySlide*, ...) with biomaterials (*LabeledExtract*, *Label*, *Sample*, ...), and all array production and biomaterial handling is tracked providing a LIMS system for arrays and biological material. The upper part of the figure shows many objects needed to support the basic microarray experimentation flow; the *Reporter *module provides information about probes on an array, *Annotaions *provides annotation information to basically any item in BASE, *Quota *handling, and *Plugins *&* Job *handling for running analysis on a cluster of back-end computers. These utility modules are connected with the lower part modules but these connections are excluded in the figure to avoid clutter. For further details on the different modules and interconnections please read the developer documentation available at the BASE web site http://base.thep.lu.se.

The BASE application features many components; MIAME compliance, multi-user, data sharing, data access management, array and biomaterial LIMS, multiple array platforms, extensibility, configurable plug-ins, annotation customisation, streamlined access to analysis tools, integration of MEV and GenePattern, web services API, and more. To support all components the underlying relational database has grown to become very large and complex, especially since BASE itself works with objects posing additional database tables to keep track of objects stored in a relational database. In Figure 1 we present an overview over the BASE data layer modules and how they interact with each other. Rather than trying to describe every module in detail we highlight some of the more important features made possible by the underlying BASE design.

### Information and annotation management

BASE features a biomaterial LIMS tracking biological material from its source to hybridisation and ultimately to raw data and analysis. All events throughout sample handling are tracked and information on used and remaining quantities, physical sample locations, quality control information, and sample relations is stored in BASE. Plates with biomaterial can be created and plate events are easily performed for extraction or labelling events. Although becoming less commonly used the array production LIMS of BASE1 is retained to support researchers with spotting facilities, e.g., protein array production and BAC array printing that may not be commercially available.

Events in biomaterial and array LIMS are annotable with protocols and event dates, and most items can be annotated with customisable annotation types such as floats, integers, dates, and Boolean flags. Annotations are either free form or from a preset list of values, and can be marked as required for MIAME compliance. The annotation system is searchable and the user can select any annotations to be experimental factors in analysis.

### Data sharing and privacy

One of the important features of BASE is its usefulness as a local data repository. The repository functionality is amended with data grouping, sharing, and privacy policies. A BASE *project *is used to group items (biomaterial, hybridisation, raw data, and experiments) into a logical entity, and a BASE *experiment *is a collection of bioassays (array data) grouped logically together for further analysis. All items can co-exist in several *projects *and *experiments *without any unnecessary copying of information. 

Data privacy is guarded by the data owner and BASE allows the owner to set data access rules. To this end, each item in BASE is owned by a user enabling him to share data with colleagues. The grouping of data in projects allows the data owner to simply include other users in a project in order to share data. Each item can have different access levels even within a project, and project members can have different privileges. The data access rules are very flexible and can be overwhelming since access levels on almost any item can be individually set. However, using projects, the proper access levels can be set at a single point of interaction.

### File and directory structure

BASE has an integrated file system to provide a possibility for researchers to collect all data files related to a project in one single storage location. Data files are uploaded using a web browser or an ftp client. The file storage is an integral part of a strategy to store all microarray experimentation relevant data in BASE, even data types not already supported in analysis. Collecting all data allows future reuse of the data as more data are produced, and new analysis tools becomes available.

### Supported platforms

There are many types of microarrays, techniques, and brands available for researchers; one- or two-channel hybridizations, spotted cDNA/oligo arrays, Affymetrix (GeneChip), Illumina (SNP, DASL, WGEX, microRNA), aCGH, SNP, tiling arrays, and many more. Data are produced in different file formats that must be treated differently depending on type. Nevertheless *any *type of microarray data can be stored in BASE.

Today, many platforms and experimental setups are supported in downstream analysis but some microarray techniques cannot currently be analysed within BASE simply because lack of support in available plug-ins. The problem is resolved by creating new, or extending available, plug-ins that add analysis capabilities of platforms and techniques not readily supported in analysis. Extending analysis capabilities to new technologies is only a matter of local needs and resources. We add support for platforms in use at the Lund University microarray facility and make our tools freely available to the community. Even though analysis of all technologies is not readily supported in BASE the repository feature merits use of BASE.

### Analysis, extensions, and plug-ins

BASE features a hierarchically organised analysis interface that allows data filtering, normalisation, transformation, and other analyses. Parameters and settings are automatically stored for each step in the analysis. The selection of analysis tools depends on array type and available plug-ins where a wide range of tools are pre-installed with BASE, and optional plug-ins can be downloaded from the BASE plug-in site http://baseplugins.thep.lu.se. BASE capitalise from other software tools, such as MEV and GenePattern, by integrating them into the user interface. Such integrations provide streamlined access to analysis modules in external tools. BASE even features a rudimentary manual transform creator that enables researchers to add analysis steps within the hierarchical overview of analysis performed independently of BASE. The transform creator enables storage of result files and parameter information for archival, tracking, and sharing purposes.

The analysis of microarray data is continuously evolving with new methods and techniques. To this end BASE provides extensions and plug-in programming interfaces (APIs) to enable straightforward additions of new analysis tools. The use of the APIs is well documented and there are numerous examples on how to create extensions. The MEV, GenePattern, and ftp-server integrations all utilise the extension mechanism, and the automatically generated overview plots available in the experimental analysis view are also extensions. The plug-in API is used for all data imports and exports, and most analysis tools, providing new developers a lot of example code to examine when they create BASE plug-ins.

### Batch upload and download of data

File, annotation, and item upload can be done asynchronously as data are generated or information becomes available. To relieve researchers from the tedious task of entering data one by one a set of batch import tools was created. The information generated throughout the experimental work is uploaded to BASE in plain tab-separated files. These files are supplied to batch importer plug-ins that parse the files and create items and associations according to the information in the files. The same plug-ins can be used to batch update many items. Similarly, annotating items is done by creating tab-separated files with annotation information, uploading these to BASE, and loading the file content into the database using annotation importers. If needed, annotations are easily updated with the same mechanism.

Files uploaded to BASE are stored in the directory structure within BASE and multiple files are easily transferred to BASE either packaged in compressed files with a single upload action, or by using an ftp client supporting transfer of file structures. Similarly, downloading multiple files is straightforward either using an ftp client or by a single click in the BASE web interface. Download of items is done through item listing views enabling users to filter and select what information should be downloaded.

### Repository and standards

The Microarray Gene Expression Data Society (MGED) develops and maintains standards for data acquisition, representation, and interchange such as the MIAME guidelines [2], the MAGE-TAB interchange format [10], and the MGED Ontology for microarray experiments [11]. BASE does not enforce the use of the MGED standards but support storage of information required by MIAME. BASE has an experiment item overview functionality useful for validating information related to experiments. The validation level is user selectable of which the option regarding MIAME compliance is most relevant here. When users or server administrators create annotation types in BASE these annotation values can be marked as required by MIAME and optionally defined to be a list of pre-defined values from a controlled vocabulary. Validation will check for inconsistencies and report errors, and give the user an opportunity to fix issues immediately or later. After resolving the issues raised by the validation, data can be exported for submission to public repositories such as ArrayExpress [12], Gene Expression Omnibus [13], and CIBEX [14].

### Comparison with other similar tools

In parallel to the evolution of microarray technology several software solutions have been created to manage and analyse the large amounts of data generated in microarray experimentation. We have made a non-exhaustive comparison of selected software that provides both data management and analysis capabilities, are free of charge to use for non-commercial and academic use, and where active development was shared to the community within the last two years.

In Table 1 we indicate which features are available in the tools we compared. All tools are web browser applications with the exception of ArrayTrack [15] that is a Java web start enabled Java client/server application, *i*.*e*., it can automatically be started over the Internet. All tools provide user and group management, data sharing and analysis capabilities, and all but EzArray [16] are MIAME compliant. EzArray, MIMAS [17], and SBEAMS-Microarray [18] support a few array platforms each whereas ArrayTrack, BASE, and SMD [19] aim at being multi-platform supporting most of the major array techniques. MIMAS singles out as the only tool to support storage of processed ultra high-throughput DNA sequencing data together with Affymetrix data in the same database. BASE and SMD both provide spotted array production LIMS and BASE is the only program to also feature a biomaterial LIMS. Installation is straightforward for most of the tools but for SBEAMS-Microarray and SMD installation is claimed by the projects themselves to be non-trivial.

**Table 1:** Comparison of selected microarray data management software.

*Feature*	ArrayTrack	BASE	EzArray	MIMAS	SBEAMS	SMD
Affymetrix	x	x	x	x	x	x
Spotted arrays	x	x				x
Illumina	x	x		x		x
Array LIMS		x				x
Biomaterial LIMS		x				
Connectivity to analysis and visualization tools	x	x	x	x	x	x
Wizard-based annotation and experiment creation	x	x		x		x
Batch import of data	x	x				x
User/group management	x	x	x	x	x	x
Experimental factors definition	x	x				
Data sharing and permissions management	x	x	x	x	x	x
MIAME compliant	x	x		x	x	x
ArrayExpress/GEO		x	x	x		x
MGED Ontology support				x		

All compared tools are web browser based except for ArrayTrack which is a Java web start enabled Java application. An 'x' character denotes supported feature. See main text for a discussion about the different software.

All tools have their positive and negative traits depending on what the tools try to achieve. The tool should carefully be selected to fit current and future needs, and on the expected development and maintenance of the software.

## Conclusion

BASE is an annotable microarray data repository and analysis application providing researchers with efficient information management and analysis. BASE stores all microarray experiment related data, biomaterial information, and annotations regardless if analysis tools for specific techniques or data formats are readily available. As new techniques becomes available software applications should be expendable and modifiable to support changed needs. The BASE team is committed to continue improving and extending BASE to make it usable for even more experimental setups and techniques, and we encourage other groups to target their specific needs leveraging on the infrastructure supplied by BASE.

## Availability and Requirements

BASE is GNU Public License version 3 [20] licensed open source software. All software required to run BASE is freely available. Users can use any standards compliant web browser to access BASE, and a demonstration server is available for anyone who wants to try BASE before installing it locally.

**Project name: **BASE

**Web site: **http://base.thep.lu.se

**Demo server: **http://base2.thep.lu.se/demo

**Operating systems: **Platform independent

**Programming language: **Java version 6

**Other requirements: **Apache Tomcat servlet container version 6, MySQL or PostgreSQL.

**License: **GNU Public License version 3

## Authors' contributions

JVC participated in the design of the presented software application. NN is the lead software developer, designed and implemented the software and plug-ins. MS implemented the software and plug-ins. JH participated in design of the application, implemented plug-ins, and coordinates the BASE project. JVC and JH wrote the manuscript. All authors read and approved the final manuscript.
